# Micellar Form of a Ferrocene-Containing Camphor Sulfonamide with Improved Aqueous Solubility and Tumor Curing Potential

**DOI:** 10.3390/pharmaceutics15030791

**Published:** 2023-02-27

**Authors:** Maria Schröder, Maria Petrova, Georgi M. Dobrikov, Georgy Grancharov, Denitsa Momekova, Petar D. Petrov, Iva Ugrinova

**Affiliations:** 1 Institute of Molecular Biology “Akad. Roumen Tsanev”, Bulgarian Academy of Sciences, Acad. G. Bonchev St., bl 21, 1113 Sofia, Bulgaria; 2 Institute of Organic Chemistry with Center of Phytochemistry, Bulgarian Academy of Sciences, Acad. G. Bonchev St., bl 9, 1113 Sofia, Bulgaria; 3 Institute of Polymers, Bulgarian Academy of Sciences, Akad. G. Bonchev St., bl 103A, 1113 Sofia, Bulgaria; 4 Faculty of Pharmacy, Medical University of Sofia, 2 Dunav St., 1000 Sofia, Bulgaria

**Keywords:** block copolymers, polymeric micelles, cancer therapy, ferrocene-based drugs

## Abstract

The discovery of new anticancer drugs with а higher, more specific activity and diminished side effects than the conventional chemotherapeutic agents is a tremendous challenge to contemporary medical research and development. To achieve a pronounced efficacy, the design of antitumor agents can combine various biologically active subunits in one molecule, which can affect different regulatory pathways in cancer cells. We recently demonstrated that a newly synthesized organometallic compound, a ferrocene-containing camphor sulfonamide (DK164), possesses promising antiproliferative activity against breast and lung cancer cells. However, it still encounters the problem of solubility in biological fluids. In this work, we describe a novel micellar form of DK164 with significantly improved solubility in aqueous medium. DK164 was embedded in biodegradable micelles based on a poly(ethylene oxide)-*b*-poly(*α-*cinnamyl-*ε-*caprolactone-*co-ε*-caprolactone)-*b*-poly(ethylene oxide) triblock copolymer (PEO_113_-*b*-P(CyCL_3_-*co*-CL_46_)-*b*-PEO_113_), and the physicochemical parameters (size, size distribution, zeta potential, encapsulation efficiency) and biological activity of the obtained system were studied. We used cytotoxicity assays and flow cytometry to determine the type of cell death, as well as immunocytochemistry to assess the influence of the encapsulated drug on the dynamics of cellular key proteins (p53 and NFkB) and the process of autophagy. According to our results, the micellar form of the organometallic ferrocene derivate (DK164-NP) exhibited several advantages compared to the free substance, such as higher metabolic stability, better cellular uptake, improved bioavailability, and long-term activity, maintaining nearly the same biological activity and anticancer properties of the drug.

## 1. Introduction

Cancer is the second-leading cause of death in the world [1]. The cancer burden continues to grow globally, exerting tremendous physical, emotional, and financial strains on both social and health systems. Along with surgery, irradiation, and immunotherapy, the most commonly used approach for treating cancer is chemotherapy, which applies different cytotoxic substances causing cell death in cancer tissue [2]. Unfortunately, these chemotherapeutic drugs have many disadvantages, including a short circulation half-life, restricted targeting, and low specificity, and can cause damage to both healthy and cancerous cells [3,4,5]. Consequently, most drug development efforts are focused on novel cancer therapeutics with improved properties.

Since the discovery of the potent anticancer activity of cis-platin [6,7], numerous comparable metal coordination complexes have been proposed [8]. Specifically, organometallic compounds are considered to be more stable, because of the stronger covalent character of their metal–ligand bond [9]. In the last decades, bioorganometallic chemistry has quickly advanced and is now closely related to medicinal chemistry, particularly with regard to metallodrugs. Among them, ferrocene derivatives have attracted much attention as potent biological agents and promising therapeutic candidates [10,11,12,13,14]. Due to their intrinsic stability in air, heat, and light, cost-effectiveness, redox characteristics, and low cytotoxicity (iron is a natural participant in intracellular metabolism), ferrocene derivatives have been considered very advantageous molecular systems [15]. This motivated our research group to conduct an extensive collaborative work involving the conception and characterization of novel ferrocene conjugates, combining different biologically active moieties with synergistic action in one molecule. Among the various ferrocene complexes that were synthesized and examined for their anticancer properties [16], one of them, a ferrocene-containing camphor sulfonamide, DK164, was singled out [17]. In comprehensive biological experiments, we found that DK164 has a remarkable cytotoxic effect on cancer cells (MDA-MB-231, MCF-7, A549, H1299) but is more tolerated by non-cancerous cells (MCF-10A, MRC5) [18]. Our data also showed the cytostatic and antiproliferative effects of the compound on cancer cells and its pro-apoptotic properties. Furthermore, we demonstrated the impact of DK164 on the process of autophagy, on the cellular localization of the key proteins p53 and NFkB, and on the redox-balance in cancer cells; thus, we gave first insight into its molecular mechanism of action [17,18]. Although DK164 appears to be a promising potential chemotherapeutic, it still encounters the problems of poor solubility in physiological fluids and low bioavailability, which are common for the lipophilic ferrocene derivatives. To overcome this issue, we applied a nanotechnology-based approach, which allowed us to increase drug solubility by using polymeric nanocarriers.

In the last decades, the interest of using nanoparticles for targeted delivery of therapeutic and diagnostic substances has risen [19,20,21,22,23]. Since nanomedicine is expected to boost the success in the treatment of cancer, numerous synthetic polymers have been used for developing intelligent, tailor-made drug delivery systems that can enhance the pharmacokinetics of poorly water-soluble drugs and thus improve cancer treatment effectiveness and reduce the side effects of conventional therapies [24,25,26,27,28,29,30]. The unique physicochemical characteristics of nanocarriers, such as small size, in vivo stability, and (multi)functionality, enable them to transport drugs effectively, target cancer tissue selectively, and regulate drug release. In addition, nanometric agents preferentially reach the tumor site and accumulate in cancer tissue with leaky vasculature, a process known as enhanced permeability and retention effect (EPR) [31]. Polymeric micelles are a kind of self-assembled nanoparticles (NPs), comprising a hydrophilic exterior layer and a hydrophobic core, which are able to transport hydrophobic drugs in vivo [32]. In addition, these NPs can be fabricated from biocompatible and nontoxic polymers, which makes them safe for use. Their favorable dimensions enable the progressive administration of medications while preventing immune system response and spleen epithelial cell filtration.

In this work, we describe the development of a novel micellar form of DK164 by embedding the drug in biodegradable micelles based on PEO_113_-*b*-P(CyCL_3_-*co*-CL_46_)-*b*-PEO_113_ triblock copolymer. The main physicochemical characteristics and the biological activity of DK164-NP were assessed as well.

## 2. Materials and Methods

### 2.1. Materials

ε-Caprolactone (99%, Alfa Aesar, Haverhill, MA, USA), tin(II) trifluoromethanesulfonate (98%, Acros, Geel, Belgium), heptane (99%, Fisher Chemicals, Hampton, NH, USA), acetone (99.5%, Fisher Chemicals), 4-pentynoic acid (95%, Acros), cinnamyl chloride (95%, Acros), ethyl acetate (99.5%, Fisher Chemicals), and N,N-diisopropylethylamine (99+, Acros) were purchased from Labimex, Bulgaria, and used as received. α-Bromoisobutyryl bromide (98%, Sigma–Aldrich, St. Louis, MO, USA), triethylamine (≥99%, Fluka), copper (I) bromide (98%, Sigma–Aldrich), N-(3-dimethylaminopropyl)-N’-ethylcarbodiimide hydrochloride (98%, Merck, Rahway, NJ, USA), lithium diisopropyl amide (1.0 M in THF/hexanes, Sigma–Aldrich), N,N,N′,N′,N″-pentamethyldiethylenetriamine (99%, Sigma–Aldrich), hexamethylphosphoramide (99%, Sigma–Aldrich), propargyl bromide (80% in toluene, Sigma–Aldrich), ammonium chloride (99.5%, Honeywell, Charlotte, NC, USA), sodium azide (99+, Fluka), 4-dimethylaminopyridine (99+%, Sigma–Aldrich), butanediol (99%, Merck), and silica gel (SiO_2_, 70–230 mesh, Merck) were purchased from FOT, Bulgaria, and used without purification. Methoxy poly(ethylene glycol) (PEO_113_-OH, MW 5000 g·mol^−1^, Fluka, FOT, Bulgaria) were precipitated in cold methanol (−40 °C), filtered and dried under vacuum at 40 °C overnight. A549 and H1299 human lung carcinoma cell lines and MRC5 non-cancerous lung fibroblasts (from the ATTC collection of cell cultures) were purchased from LGC Limited, Augsburg, Germany. Cell culture-related reagents were: 0.25% Trypsin-EDTA (1×), Phosphate Buffered Saline (PBS), Dulbecco’s Modified Eagles Medium (DMEM), F12K and RPMI-1640 media, Penicillin-Streptomycin antibiotic, and Fetal Bovine Serum (FBS), and were purchased from Thermo Fisher Scientific, via Antisel Bulgaria LTD. (Sofia, Bulgaria). MTT (≥98%) and DMSO (≥99.9%) were purchased from Sigma–Aldrich via FOT LTD (Sofia, Bulgaria). Annexin V Apoptosis Detection Kit FITC/PI was purchased from Thermo Fisher Scientific, via Antisel Bulgaria LTD. (Sofia, Bulgaria). Primary antibodies: polyclonal rabbit anti-LC3 (ab51520) and antiNFkB (ab16502) were obtained from Abcam, (Cambridge, UK); monoclonal mouse anti-p53 (645802) and monoclonal mouse anti-vimentin (677802) were received from Biolegend, (San Diego, CA, USA). Secondary antibodies, the donkey anti-mouse Alexa 555 antibody (A-31570), goat anti-rabbit Alexa 488 antibody (A-11001), and ProLong Diamond mounting media (P36961) were obtained from Thermo Fisher Scientific, via Antisel Bulgaria LTD. (Sofia, Bulgaria).

### 2.2. Synthesis of DK164 and Triblock Copolymer

Ferrocene derivative DK164 was synthesized in our previous work [16], starting from commercially available (1*S*)-(+)-10-camphorsulfonyl chloride. More details about synthetic procedures and the characterization of this compound are presented in Supplementary Information (Figure S1).

The synthesis of PEO_113_-*b*-P(CyCL_3_-*co*-CL_46_)-*b*-PEO_113_ triblock copolymer has been reported elsewhere [33]. The preparation steps, described in detail in the SI (see Figure S2), are summarized as follows: the hydrophobic P(CyCL_3_-*co*-CL_46_) precursor was obtained by (i) ring-opening copolymerization of ε-caprolactone and α-propargyl-ε-caprolactone; (ii) grafting of cinnamyl moieties via “click” reaction; and (iii) functionalization with terminal azide groups. Mono-alkyn functional poly(ethylene oxide) macroreagent, PEO_113_-C≡CH, was prepared by a carbodiimide catalyzed reaction of OH-PEO_113_-CH_3_ with 4-pentynoic acid. The amphiphilic triblock copolymer was synthesized by a “click” coupling reaction of PEO_113_-C≡CH (4 eq.) and N_3_-P(CyCL_3_-*co*-CL_46_)-N_3_ (1 eq.). M_n_^1H-NMR^ = 15,700 g·mol^−1^; M_n_^GPC^ = 8800 g·mol^−1^; M_w_/M_n_ = 1.13. NMR spectra of the precursors (Figure S3) and the NMR spectrum (Figure S4) and GPC chromatograms (Figure S5) of the triblock copolymer are shown in the SI.

### 2.3. Analysis of Copolymer and Nanoparticles

Proton Nuclear Magnetic Resonance (^1^H-NMR) spectra were recorded using a Bruker Avance II+600 apparatus with solvent CDCl_3_. Gel Permeation Chromatography (GPC) analysis were carried out on а HPLC system (Shimadzu Nexera), equipped with a RI detector, an autosampler, a degasser, a pump, and three PSS SDV columns (5 µm Linear M; 5 µm, 100 Å; and 5 µm, 50 Å) at temperature of 40 °C and sample concentration of 1 gL^−1^. Tetrahydrofuran (HPLC grade) was used as the eluent at a flow rate of 1.0 mL min^−1^. Poly(ethylene oxide) standards were used for the calibration of the instrument. The hydrodynamic diameter, dispersity index and zeta potential of blank and drug-loaded micelles were determined by using a ZetaSizer NanoZS (Malvern Instruments, Malvern, UK), instrument, equipped with a 633 nm laser. The measurements were conducted at a scattering angle of 175° and a temperature of 37 °C. Each sample was measured three times. The UV-vis absorption spectra of DK164-NP samples were recorded on a Shimadzu UV-1800 UV-vis spectrophotometer, using quartz cells (path length 1 cm).

### 2.4. Preparation of Micellar Nanoparticles

50 mg of the copolymer were dissolved in 10 mL of acetone, and then the organic solution was added dropwise to 25 mL of distilled water at 25 °C, under stirring (1000 rpm). Next, the organic solvent and 3 mL of water were evaporated under vacuum at 40 °C, giving a colorless aqueous micellar solution with a concentration of 2.273 g/L.

### 2.5. Drug Loading and In Vitro Release

A solution of 0.625 mg DK164 in 2 mL of ethanol was added dropwise to 5.5 mL aqueous solution of micelles. The mixture was stirred for 30 min, and ethanol evaporated in vacuo at 40 °C to form a stable aqueous colloid (mass ratio carrier/drug = 20:1).

The obtained sample was filtered through a nylon filter (0.45 μm). The filter was rinsed with ethanol to collect the non-encapsulated DK164. UV spectroscopy (λ = 318 nm) was used to determine the amount of free DK164. A pre-build calibration curve was used, with linearity in the concentration range of 0.04 to 20 µg/mL and correlation coefficient R^2^ = 0.997. The encapsulation efficiency (EE) was calculated as follows:

(1) $$\mathrm{EE}\left(\%\right)=\frac{\mathrm{Total}\;\mathrm{mass}\;\mathrm{of}\;\mathrm{DK}164-\mathrm{Mass}\;\mathrm{of}\;\mathrm{free}\;\mathrm{DK}164}{\mathrm{Total}\;\mathrm{mass}\;\mathrm{of}\;\mathrm{DK}164}\times 100$$

The in vitro drug release of DK164 from the micellar nanoparticles was determined using the dialysis method. In brief, 1 mL samples of DK164-loaded polymer micelles (equal to 0.1 mg/mL DK164) were placed in dialysis bags (Spectra/Por™, Spectrum Labs, Arden, NC, USA) with a molecular weight cut-off of 10,000 Da. The dialysis bags were immersed in a 50 mL phosphate-buffered saline (pH 7.4) containing 10% (*w*/*v*) ethanol at 37 ± 0.5 °C under constant stirring at 100 rpm. At pre-determined time intervals, 1 mL of acceptor medium was withdrawn and replaced with fresh medium. The amount of released DK164 was measured using a UV–Vis spectrophotometer at 318 nm, and the cumulative release (Q%) was calculated. The release experiment was performed in triplicate, and the results were expressed as a mean value ± SD. The diffusion of free DK-164 (dissolved in ethanol) through the dialysis tube was assessed at a concentration equal to the drug concentration into the micellar formulation under the same experimental conditions.

### 2.6. Cell Culture

The complete medium for lung cancer cell line A549 was F12K and for H1299—RPMI-1640, supplemented with 10% heat-inactivated fetal bovine serum, 100 µg/mL streptomycin, and 100 units/mL penicillin. MRC5 cells were cultured in Minimum Essential Medium supplemented with 10% heat-inactivated fetal bovine serum, 100 units/mL penicillin, and 100 µg/mL streptomycin. Cells were maintained in an air-humidified incubator, in 5% CO_2_ at 37 °C. Only cells growing in the exponential phase were used in the experiments.

### 2.7. In Vitro Cytotoxicity Assay

The growth inhibitory effects of the obtained micellar formulation DK164-NP on the human lung cancer cell lines A549 and H1299 and the non-cancerous lung fibroblasts MRC5 were assessed by the MTT (3-(4,5-dimethylthiazol-2-yl)-2,5-diphenyl tetrazolium bromide) assay method, in which the metabolically active cells reduce MTT to a DMSO soluble formazan product that can be analyzed colorimetrically [34]. Briefly, cells were seeded at 3 × 10^3^ per well in quadruplicates in 96-well flat-bottom plates and pre-incubated for 24 h (at 37 °C and 5% CO_2_) to permit them to attach. Cells were treated with the free substance DK164 (dissolved in max. 0.5% DMSO) or the micellar form DK164-NP at concentrations for DK164 ranging from 6 µM to 100 µM. The micellar solution of the used block copolymers was tested in a corresponding dilution to evaluate the nanocarrier’s cytotoxicity. After 72 h of incubation, MTT solution (500 μg/mL) was added, and samples were incubated for additional 3 h. Next, the yellow formazan crystals, formed in each well, were dissolved with 100 μL of DMSO. The optical density was measured with a ELISA microplate reader Varioscan Lux (Thermo Fisher Scientific, Waltham, MA, USA) at a wavelength of 570 nm. Cell viability (%) was determined as the ratio of the number of living cells in treated samples to that of the control [23]. The experiments were performed in triplicate, and the results are represented as mean ± SD.

### 2.8. Cytometric Detection of Apoptosis

Cell apoptosis rate was determined by flow cytometry with Annexin V Apoptosis Detection Kit FITC/PI, according to the manufacturer’s instructions. The cells to be detected were plated in 12-well plates before treatment with the calculated IC50 concentrations of DK164-NP for 24 and 48 h. For the analysis, cells were collected, washed with cold phosphate-buffered saline (PBS), and resuspended in Annexin V Binding Buffer (AVBB) at a concentration of ~1 × 10^6^ cells/mL in 100 µL. Then, 5 µL of Annexin V–fluorescein isothiocyanate (FITC) was added, and cells were incubated on ice for 15 min. Afterward, the samples were washed with AVBB, resuspended in 200 µL AVBB containing PI, and incubated on ice in the dark for 30 min. The apoptosis rate was analyzed by flow cytometry with proper compensating settings on a Becton Dickinson FACScalibur instrument (BD Biosciences, San Jose, CA, USA). The percentages of live, early apoptotic, late apoptotic, and necrotic cells were quantified with FlowJo v.10.8.1 software (BD Biosciences, Ashland, OR, USA).

### 2.9. Immunofluorescence Microscopy

To observe the autophagic marker LC3 and the cellular localization of the transcription factors p53 and NFkB cells were grown on coverslips and fixed with 3.7% (*v*/v) paraformaldehyde in PBS at room temperature for 5 min, followed by permeabilization with 0.1% (*w*/*v*) Triton-X-100 in PBS for 5 min. Next, cells were blocked with 10% (*w*/*v*) fetal calf serum, 1% BSA (*w*/*v*) and 0.1% TX-100 (*w*/*v*) in PBS for 30 min at 37 °C. Primary antibodies- polyclonal rabbit anti-LC3, monoclonal mouse anti-p53, polyclonal rabbit anti-NFkB, and monoclonal mouse anti-vimentin were diluted in PBS (1:100–1:500) and incubated for 30 min at 37 °C. For visualization, cells were incubated with secondary antibodies-goat anti-rabbit Alexa 488 antibody and donkey anti-mouse Alexa 555 antibody, at 1:2000 dilution at 37 °C for 30 min. Finally, the coverslips were mounted in ProLong Diamond mounting media containing 400 ng/mL DAPI for nucleus staining. Images were acquired in Zeiss AxioVert 200 M microscope using a 63× objective lens (NA = 1.4), equipped with a CCD camera Axio Cam MRm. Laser intensities and detector gains were maintained at the same level during all imaging sessions. Image processing and calculations were performed by using ImageJ and Cell Profiler cell image analysis software (Broad Institute’s Imaging Platform, Cambridge, MA, USA).

### 2.10. Statistical Analysis

All in vitro experiments were repeated at least three times. Data were analyzed using Microsoft Excel and GraphPad Prism v. 8.0 (Dotmatics, San Diego, CA, USA) and are shown as mean ± SD. A statistically significant difference was considered at *p* < 0.05 using unpaired Student′s *t*-test or two-way ANOVA analysis with Tukey’s multiple comparison tests of variance for two and multiple sample comparisons, respectively.

## 3. Results

### 3.1. Preparation and Characterization of Blank and Drug-Loaded NPs

**Figure 1:**
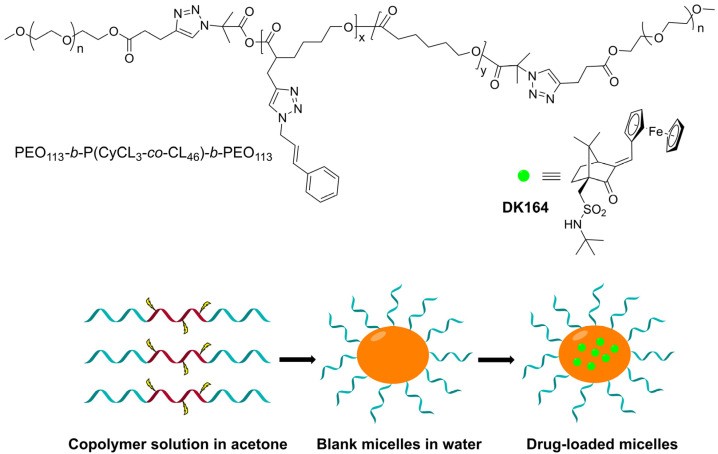
Preparation of DK164-loaded micellar nanoparticles based on PEO_113_-*b*-P(CyCL_3_-*co*-CL_46_)-*b*-PEO_113_ amphiphilic triblock copolymer.

The micellar nanoparticles were prepared by the solvent evaporation method. In the first step, PEO_113_-*b*-P(CyCL_3_-*co*-CL_46_)-*b*-PEO_113_ was dissolved in acetone, a common solvent for PEO and P(CyCL-*co*-CL), and then the solution was added to water. The organic solvent was removed by evaporation, thus forming a stable aqueous micellar solution. DK164 was loaded into the pre-formed micelles by adding an ethanol solution of the drug to the micellar solution and subsequent evaporation of the ethanol (Figure 1).

**Figure 2:**
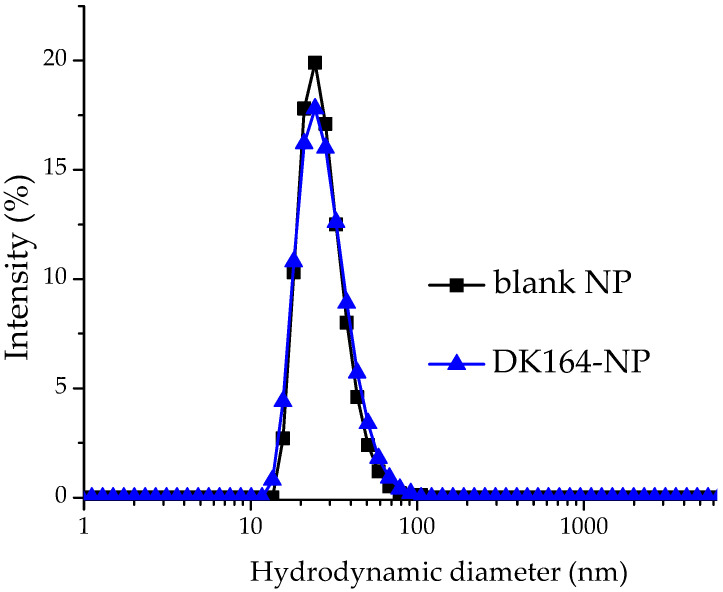
Hydrodynamic diameter distribution of blank and drug-loaded micelles.

The blank and DK164-loaded micelles were analyzed by dynamic and electrophoretic light scattering. The intensity-weighted size distribution plot of the blank micelles was monomodal and relatively narrow (Figure 2).

The calculated values of hydrodynamic diameter (D_h_), dispersity index (DI) and zeta potential are listed in Table 1. The polymeric carriers possess nanoscopic dimensions and nearly neutral surface charge. Expectedly, embedding DK164 into the micellar core did not alter the main physicochemical characteristics of the particles.

### 3.2. Drug Release Study

**Figure 3:**
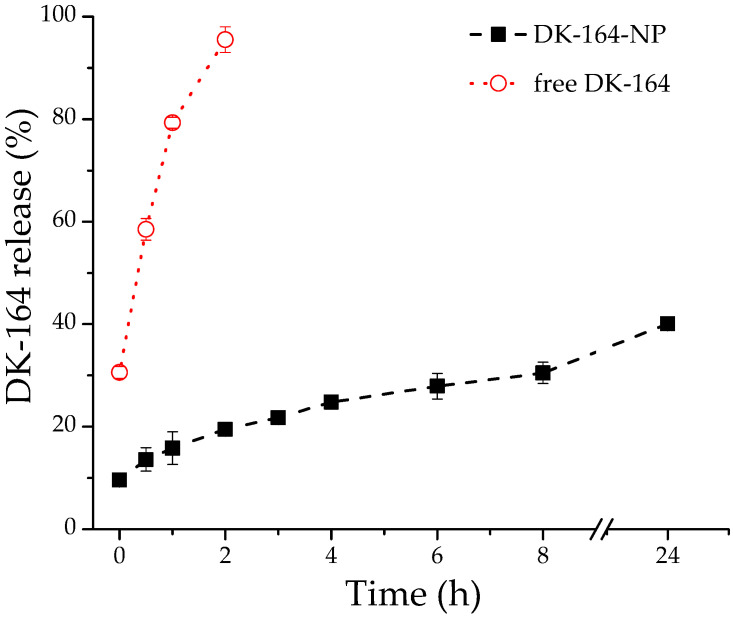
Release profile of DK164 from NPs in PBS with 10% ethanol solution at 37 °C (black line). The red line refers to the diffusion of pure DK-164 (applied as ethanol solution) through the membrane. Each point represents average ± SD (n = 3), and the lines are just a guide for the eye.

The in vitro DK164 release from micellar nanocarriers was evaluated by regular dialysis technique against 50 mL PBS (pH 7.4), with the presence of 10% ethanol maintaining the sink conditions. As evident from Figure 3, approximately 42% of the encapsulated agent was released at the 24th hour and, more importantly, no initial burst release was observed. To confirm that the observed slow release of DK164 is due to a controlled diffusion through the polymeric core of the micelles and not from the dialysis membrane itself, we also investigated the release profile of free DK164, administered as an ethanolic solution, under the same experimental conditions. As can be seen from Figure S3, the non-formulated DK164 passed through the dialysis membrane in 2 h. These findings indicate that the elaborated micellar nanocarriers are able to release DK164 in a sustainable manner for a prolonged period. The observed sustained-release characteristics are probably due to the hydrophobic interactions between the micellar core and the highly hydrophobic DK164.

For more detailed characterization of the release mechanism of DK164 from the prepared micelles, the data obtained were fitted to the main kinetic models: zero-order kinetics, first-order kinetics, the Higuchi model and the Korsmeyer–Peppas model. The Korsmeyer–Pepas model was determined as the most appropriate kinetic model describing the release process of DK164 with R^2^ values of 0.996 (Table S1 and Figure S6 in SI), indicating that drug release follows the Fickian diffusion mechanism, where DK164 release is controlled by the stable micellar core [35].

### 3.3. Evaluation of the In Vitro Cytotoxicity of DK164-NP

**Figure 4:**
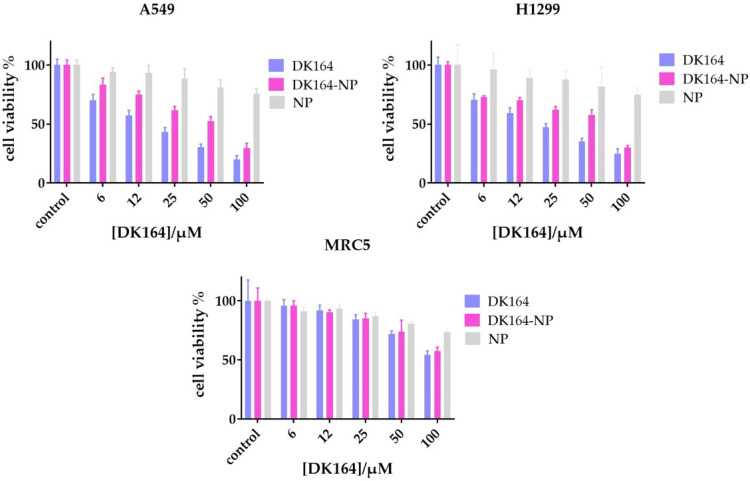
Cell viability assay: cancerous (A549, H1299) and non-cancerous cells (MRC5) were treated with various concentrations of the free compound DK164, the micellar form of the compound (DK164-NP), and the corresponding amount of used copolymers (NP) for 72 h and the percentage of cell viability was calculated in comparison with control. Data are represented in mean ± SD.

The results of the cytotoxicity tests (Figure 4) showed that DK164-NP was biologically active, and its effect on the tumor cell lines A549 and H1299 was similar but slightly weaker than that of the free substance. In the non-cancerous control cell line MRC5, the micellar form showed much similar inhibitory activity, but most importantly it retained the selectivity characteristic of the pure substance DK164. As this selectivity was one of the most promising properties of the substance, we can conclude that the micellar system DK164-NP should be further characterized biologically. The pure copolymer weakly affected the cell viability in the highest concentration; as this effect resulted in more than 70% viable cells for all tested cell lines, the copolymer could be considered non-toxic.

### 3.4. Flow Cytometry Analysis of Apoptosis

**Figure 5:**
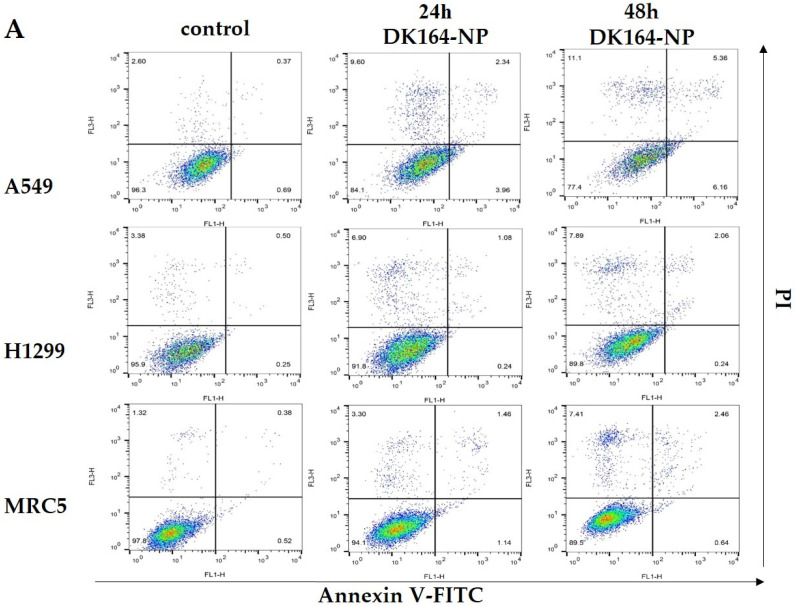

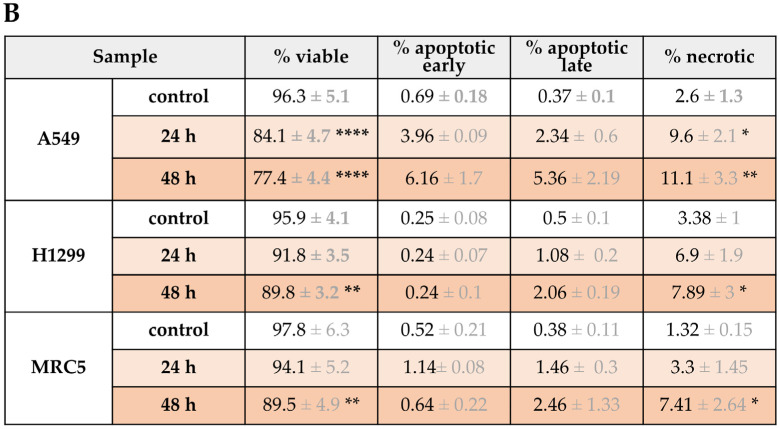
Cell apoptosis: (**A**) Flow cytometry histograms of A549, H1299 and MRC5 cells, treated with the calculated IC50 concentrations of DK164-NP for 24 h or 48 h and then stained with Annexin V-FITC and Propidium Iodide (PI). The four quadrants in each panel correspond, respectively, to: necrotic cells (upper left); late apoptotic cells (upper right); early apoptotic cells (low right); viable cells (low left); (**B**) Quantitative analysis of the flow cytometry results from at least three independent experiments (n = 3) ±SD. Multiple comparisons function of two-way ANOVA (Tukey’s multiple comparison test) was used to compare the mean of treated samples with the mean of control column. Probability values were considered significant at the * *p* < 0.05, ** *p* < 0.01, **** *p* < 0.0001.

Our previous study described the potential of pure DK164 as an apoptosis inducer in A549, H1299 cancer cells, and non-cancerous MRC5 cells [18]. Here, we used the same approach to distinguish early from late apoptosis and necrosis by AnnexinV-FITC/PI method. The cells were treated for 24 h and 48 h with the calculated IC50 concentrations of DK164-NP and analyzed by flow cytometry (Figure 5). As seen in the shown representative dot plots of these experiments (Figure 5A), FACS analyses revealed that DK164-NP caused apoptosis and necrosis in all three lung cell lines, similarly to the pure substance [18]. The effect here is weaker than those of the pure DK164. For instance, after 48 h treatment, the pure substance induced approximately 10% early apoptotic cells in A549 cells (with functional p53 proteins), whereas its micellar form, DK164-NP, caused 6%. Treatment of H1299 and MRC5 cells increased necrotic cells by 2.5 and 10 times at 48 h.

### 3.5. Influence of DK164-NP on Autophagy

**Figure 6:**
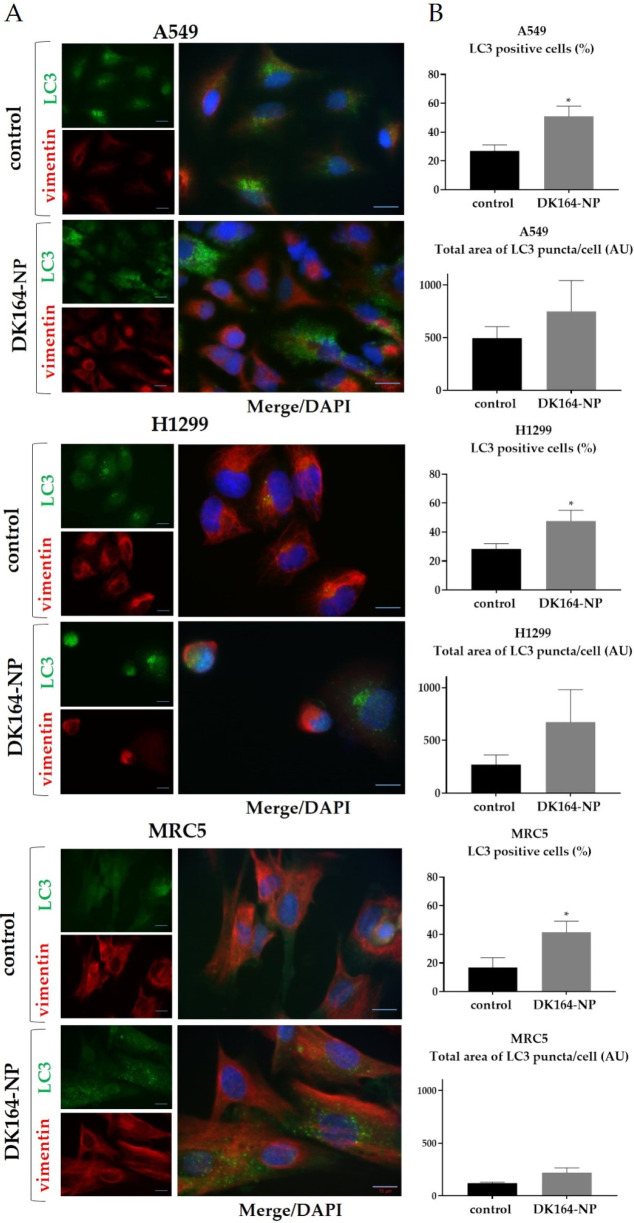
(**A**) Representative immunofluorescence images of the morphology of LC3-positive structures (green), vimentin staining (red), and DAPI-staining (blue) in A549, H1299, and MRC5 control cells and cells, treated with the calculated IC50 concentration of DK164-NP for 24 h; (**B**) Quantification of the LC3 positive cells and the LC3 average total area per cell from the images in A performed using ImageJ quantification tool (* *p* < 0.05). Quantification was performed from three experiments with >75 cells quantified for each condition. Error bars represent standard deviation (SD); AU: arbitrary units. Scale bars represent a distance of 15 µm.

Autophagy is a highly regulated homeostatic process. The modulation of autophagy plays a dual role in tumor suppression and promotion, as it has complicated and often competing roles in many cancers [36,37]. The model substrate usually used for autophagy detection is the LC3 protein (microtubule-associated protein 1A/1B-light chain 3) [38]. To study the level of autophagy in A549, H1299, and MRC5 lines, caused by DK164-NP for 24 h, we performed immunocytochemistry to monitor LC3 puncta formation. Vimentin was used to visualize the cellular shape, and the nuclei were co-stained with DAPI (Figure 6A). In control, untreated cells, the LC3 signal was comparatively dim, diffused, and evenly distributed in the cytoplasm. In the LC3-positive cells, characteristic dot-shaped fluorescent spots (LC3 puncta) were observed, marking the formed LC3-II-phosphatidylethanolamine complexes.

To quantify the LC3-positive structures, we used two parameters with an average total area of LC3 puncta per cell and the percent of LC3-positive cells using the ImageJ quantification tool (* *p* < 0.05). The graphs were generated by GraphPad Prism v.8.0 software (Dotmatics, San Diego, CA, USA) (Figure 6B). Our previous work showed that pure DK164 affected breast and lung cancer cells’ autophagy [17,18]. DK164 enhanced the autophagy signals in all three lung cell lines, leading to an increase in the number of cells with active autophagy, causing an approximately 2.8-fold increase in the fraction of LC3-positive cells in A549 and H1299. Although DK164-NP increased the number of LC3-positive cells, the effect was less prominent, approximating 2-fold.

### 3.6. Effect of DK164-NP on the Dynamics of the Transcription Factors p53 and NFkB

**Figure 7:**
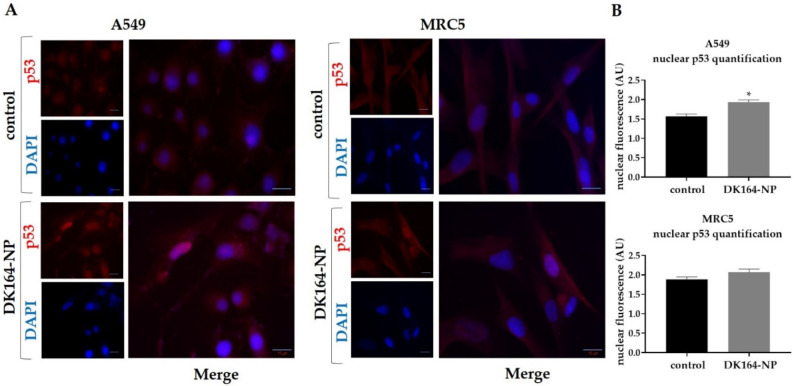
Activation and nuclear translocation of the p53 protein in A549 and MRC5 cells. (**A**) Representative immunofluorescence images of A549 and MRC5 control cells and cells treated with the calculated IC50 concentrations of DK164-NP for 48 h and labeled with anti-p53 antibody (red). DNA was co-stained with DAPI (blue); (**B**) Quantitative determination of the intensity of the nuclear p53-signal, normalized with nuclear DNA content, was performed by CellProfiler cell image analysis software (Broad Institute’s Imaging Platform, Cambridge, MA, USA) and GraphPad Prism 8.0 (Dotmatics, San Diego, CA, USA). A *p*-value < 0.05 was considered statistically significant, * *p* < 0.05. Quantification is based on three independent experiments with >50 cells scored for each condition. Error bars represent standard deviation (SD); AU: arbitrary unit. Scale bars represent a distance of 15 µm.

**Figure 8:**
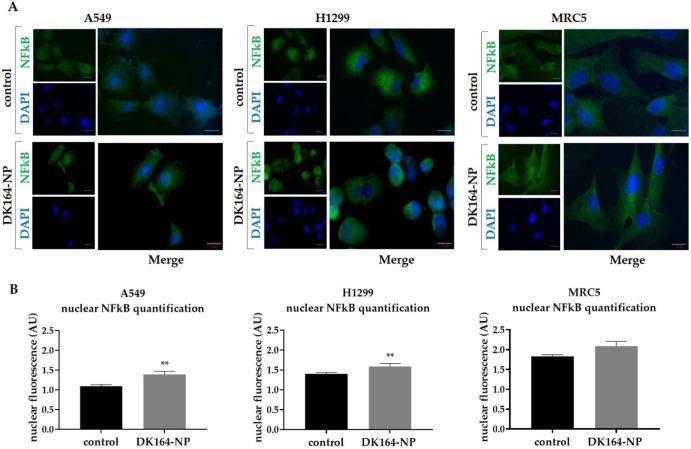
Activation and nuclear translocation of the NFkB protein in lung cells. (**A**) Representative immunofluorescent images of A549, H1299, and MRC5 control cells and cells treated with the calculated IC50 concentrations of DK164-NP for 48 h and labeled with anti-NFkB antibody (green). DNA was co-stained with DAPI (blue); (**B**) Quantitative determination of the intensity of the nuclear NFkB-signal, normalized with nuclear DNA content, was performed by CellProfiler cell image analysis software (Broad Institute´s Imaging Platform, Cambridge, MA, USA) and GraphPad Prism v.8.0 (Dotmatics, San Diego, CA, USA). A *p*-value < 0.05 was considered statistically significant, ** *p* < 0.01. Quantification is based on three independent experiments with >50 cells scored for each condition. The error bars represent standard deviation (SD). AU: arbitrary unit. Scale bars represent a distance of 15 µm.

In response to cellular stress, the p53 tumor suppressor protein is stabilized due to the inactivation of ubiquitin-mediated degradation. As a result, the protein rapidly accumulates in the nucleus and acts as a transcription factor. In response to cellular stress another group of proteins, the members of the NF-kappaB family of transcription factors translocate in the nucleus and cause transcriptional activation of anti-apoptotic genes. To monitor the dynamics of the p53 and NFκB proteins, A549, H1299, and MRC5 cells were treated with IC50 concentrations of DK164-NP for 48 h. After treatment, cover slides were incubated with antibodies against p53 or NFκB, and nuclei were co-stained with DAPI. The observations are presented in Figure 7 and Figure 8, respectively. As H1299 cells do not express the p53 protein, they were excluded from the experiment. Like the pure DK164, DK164-NP caused translocation of the p53 protein in the nucleus of A549 cells but not in non-cancerous MRC5 cells. The nuclear signal intensity in A549 increased by approximately 20% after 48 h of treatment. The treatment of A549, H1299, and MRC5 cells increased the fluorescence signal of the NFκB protein in the nucleus in all three cell lines. The effect was most prominent in A549, whereas in MRC5 it was not estimated as statistically significant.

## 4. Discussion

DK164 is a promising novel chemotherapeutic candidate possessing pronounced selectivity to normal human cells. Due to its lipophilic nature, the in vivo application of pure DK164 is useless, as the solubility of the drug in physiological fluids is limited. Developing a new drug form by embedding the hydrophobic DK164 into nanosized polymeric carriers provides an opportunity to improve its bioavailability and therapeutic efficacy. Our initial attempts to solubilize DK164 in water with the aid of some commercially available copolymers, such as poly(ethylene oxide)-*b*-poly(ε-caprolactone)-*b*-poly(ethylene oxide) (PEO-PCL-PEO) and poly(ethylene oxide)-*b*-poly(propylene oxide)-*b*-poly(ethylene oxide) (PEO-PPO-PEO), failed as the resulting samples are not colloidally stable at relatively low drug content (carrier/drug mass ratio 20:1) (see Figure S7 in SI). Recently, we demonstrated that the modification of the hydrophobic PCL block with pendant cinnamyl moieties enhances the compatibility between the hydrophobic bioactive substance and the micellar core and thus reduces the burst release of drug from the nanocarriers, enabling controlled drug release for a prolonged period [33]. Indeed, by using PEO_113_-*b*-P(CyCL_3_-*co*-CL_46_)-*b*-PEO_113_, we prepared a stable colloidal solution of DK164 in water that remained transparent, without any precipitate, for several weeks (Figure S8 in SI). This fact, together with the nanoscopic dimensions and spherical morphology of nanoparticles (Figure S9 in SI), high encapsulation efficiency, and sustained drug release profile make the micellar form of DK164 a promising candidate for advanced tumor therapy. 

To evaluate the biological activity of the micellar system DK164-NP, we applied some of the most commonly used methods for in vitro assessment. The MTT assay is the most widely used method to predict the chemotherapeutic efficacy of anticancer agents prior to in vivo testing. It is considered sensitive, accurate, and efficient for evaluating the cytotoxic potential of substances [39,40,41]. Several studies revealed that in vitro sensitivities are associated with in vivo tumor responses [42,43]. Cell viability is a general definition comprising the three parameters: cytotoxic, cytostatic, and antiproliferative effect. Therefore, any decline in viability might result from one or more of these three events. As we have already shown, DK164 has pronounced cytotoxic, cytostatic, and antiproliferative effects on cancer cells, so we can assume that the reduced cell viability of cells exposed to the micellar form DK164-NP is due to the conserved properties of the incorporated substance. Apoptosis is a fundamental mechanism of cell death, and its initiation is one of the major modes of action of chemotherapeutic agents [44,45,46]. More complicated is to delineate the possible role of autophagy as a target for anticancer therapy, because of its dual role following treatments, with response increasing or diminishing the anticancer effects [47,48,49,50]. Various cancer types may respond differently to anticancer medications’ effects on autophagy. Therefore, monitoring the autophagic level in response to treatment makes sense. The p53 protein confers an anticancer impact by inducing gene repair and, if the damage is irreparable, causing cancer cells to undergo apoptosis [51,52]. In this aspect, the activation of p53-signaling is a desired effect of the chemotherapeutics [53]. The NFκB pathway is one of the highly conserved signal pathways of gene transcription activation and exhibits complex apoptotic effects in various cell types [54]. Usually, its activation under stress conditions is associated with resistance to the chemotherapeutic pro-apoptotic signals [55,56], but it also renders Fas-induced pro-apoptotic effects [57]. DK164 already demonstrated remarkable antiproliferative activity, which involves, among others, the induction of apoptosis and autophagy and the activation of transcription factors p53 and NFkB [17,18]. The micellar form DK164-NP, displaying the observed physicochemical and pharmacokinetic benefits mentioned above, showed a comparable ability to decrease cell viability and activate a cellular response to cytotoxic stress in the lung cancer cell model. The studied cellular processes play a key role in tumorigenesis and are usually observed when the chemotherapeutic potential of new drug candidates must be examined. However, after the same incubation time, the biological effects induced by the formulated drug were slightly weaker, compared to those of the free compound. These results meet our expectations, as cells were exposed to lower drug concentrations due to the slower release of DK164 by the nanocarriers, as after 24 h only 40% of the substance are released (Figure 3). It is worth mentioning that the relatively reduced activity of the encapsulated compound can differ according to the type of experiment. So, we observed lower efficiency of DK164-NP in the FACS analysis compared to its potency in the cytotoxicity tests and immunocytochemistry observations. As Nguyen et al. suggest, this can be linked to the different percentages of compound released from the NPs under different experimental conditions as a function of the oily/polymeric medium and water partition coefficient [58].

## 5. Conclusions

A novel micellar form of the ferrocene-containing camphor sulfonamide DK164 was developed by embedding the hydrophobic bioactive substance in PEO_113_-*b*-P(CyCL_3_-*co*-CL_46_)-*b*-PEO_113_ triblock copolymer-based micelles. The drug-loaded micelles were colloidally stable in aqueous media while possessing high encapsulation efficacy, small hydrodynamic diameter (below 50 nm), and sustained release profiles. The bioassay data showed that the micellar form of DK164 has comparable antitumor activity to the free drug in the lung cancer cell model. The successful solubilization of DK164 and the preserved antitumor properties of the drug give us reason to conclude that the developed micellar system is a feasible nanoplatform for the delivery of organometallic, ferrocene-based drugs and can act as potential chemotherapeutic.

## Figures and Tables

**Table 1:** Physicochemical characteristics of blank and DK164-loaded micelles prepared from PEO_113_-*b*-P(CyCL_3_-*co*-CL_46_)-*b*-PEO_113_ triblock copolymer.

Sample	D_h_ (nm)	DI	ξ-Potential (mV)	EE (%)
Blank micelles	42.3 ± 4.2	0.21 ± 0.05	−0.82 ± 0.04	-
DK164-NP	44.7 ± 2.4	0.24 ± 0.06	−1.83 ± 0.40	98 ± 2

## Data Availability

Data sharing is not applicable to this article.

## Supplementary Materials

The following supporting information can be downloaded at: https://www.mdpi.com/article/10.3390/pharmaceutics15030791/s1, Figure S1: Schematic representation of the synthesis of DK164; Figure S2: Schematic representation of the synthesis of PEO113-b-P(CyCL3-co-CL46)-b-PEO113; Figure S3: 1H-NMR spectra of: (1) α,ω-dihydroxy poly(α-propargyl-ε-CL3-co-ε-CL46); (2) α,ω-dihydroxy poly(α-cinnamyl-ε-CL3-co-ε-CL46); (3) α,ω-dibromo poly(α-cinnamyl-ε-CL3-co-ε-CL46); and (4) α,ω-azide terminated poly(α-cinnamyl-ε-CL3-co-ε-CL46) in CDCl3; Figure S4: 1H-NMR spectrum of PEO113-b-P(CyCL3-co-CL46)-b-PEO113 in CDCl3; Figure S5: GPC chromatograms of: (1) N3-P(CyCL3-co-ε-CL46)-N3, (2) PEO113-C≡CH macroreagents, and (3) PEO113-b-P(CyCL3-co-CL46)-b-PEO113 triblock copolymer; Figure S6: Plot of the released amount of DK-164 versus ln t (Korsmeyer-Peppas model); Figure S7: Digital picture of water-based sample containing DK164 and PEO113-b-PCL35-b-PEO113, taken 24 h after loading the drug into the micelles; Figure S8; Digital picture of aqueous colloid of DK164 and PEO113-b-P(CyCL3-co-CL46)-b-PEO113, taken 24 h after loading the drug into the micelles; Figure S9: Cryo-TEM micrograph of PEO113-b-P(CyCL3-co-CL46)-b-PEO113 micelles in water; Table S1: Release kinetic models for DK-164-NP.
