# Supplementary material for: Micellar Form of a Ferrocene-Containing Camphor Sulfonamide with Improved Aqueous Solubility and Tumor Curing Potential
Source: Pharmaceutics. 2023 Feb 27;15(3):791. doi: 10.3390/pharmaceutics15030791 (PMC10054005; doi:10.3390/pharmaceutics15030791)
Supplement: Supplementary file 1 [file pharmaceutics-15-00791-s001.zip › pharmaceutics-2225130-supplementary.pdf]

Micellar Form of a Ferrocene-Containing Camphor Sulfonamide with Improved Aqueous Solubility and Tumor Curing Potential

Maria Schröder[1,†], Maria Petrova[1,†], Georgi M. Dobrikov[2], Georgy Grancharov[3], Denitsa Momekova[4], Petar D. Petrov[3*] and Iva Ugrinova[1*]

[1] Institute of Molecular Biology "Akad. Roumen Tsanev", Bulgarian Academy of Sciences, Acad. G. Bonchev st, bl 21, Sofia 1113, Bulgaria

[2] Institute of Organic chemistry with Center of Phytochemistry, Bulgarian Academy of Sciences, Acad. G. Bonchev st, bl 9, Sofia 1113, Bulgaria

[3] Institute of Polymers, Bulgarian Academy of Sciences, Akad. G. Bonchev st, bl 103A, Sofia 1113, Bulgaria

[4] Faculty of Pharmacy, Medical University of Sofia, 2 Dunav st, 1000 Sofia, Bulgaria

* Correspondence: ugryiva@gmail.com, Tel.: +359 887 985 463; ppetrov@polymer.bas.bg, Tel.: +359 02 979 6335

† The authors have equal contribution

Synthesis and analytical data of compounds

Synthesis of DK164

N-(*tert*-butyl)-1-((1*S*,4*R*)-7,7-dimethyl-2-oxobicyclo[2.2.1]heptan-1-yl)methanesulfonamide (**3**):

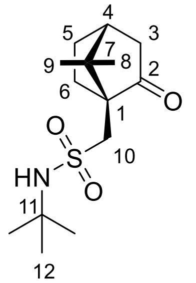

To a solution of 1.94 ml (18.35 mmol) *tert*-butylamine (**2**) in 50 ml dry DCM was added at 0°C in small portions (1*S*)-(+)-10-camphorsulfonyl chloride (**1**) as a solid (2.00 g, 7.98 mmol). The reaction mixture was stirred at r.t. for 24 h, washed with water and dried over anhydrous Na_2SO_4. The solvent was evaporated *in vacuo* and the crude product was crystallized from heptane:acetone = 10:1 ml. The crystals obtained were washed with hexane and dried *in vacuo* to give 1.57 g (69%) pure **3** as white crystals. ^{1}H NMR (600.13 MHz, $CDCl_3$, 293 K): δ = 3.49 (d, 1H, 10-H_a, *J* = 15.0 Hz), 2.99 (d, 1H, 10-H_b, *J* = 15.0 Hz), 2.40 (m, 1H, 3-H_{exo}), 2.32 (m, 1H, 6-H_{exo}), 2.12 (br t, 1H, 4-H, *J* = 4.5 Hz), 2.03 (tdd, 1H, 5-H_{exo}, *J* = 16.2, 12.1, 4.1 Hz), 1.94 (d, 1H, 3-H_{endo}, *J* = 18.7 Hz), 1.90 (m, 1H, 6-H_{endo}), 1.44 (m, 1H, 5-H_{endo}), 1.41 (s, 9H, 12-H), 1.05 (s, 3H, 9-H), 0.91 (s, 3H, 8-H). ^{13}C NMR (150.92 MHz, $CDCl_3$, 293 K): δ = 216.89 (1C, 2-C), 59.28 (1C, 1-C), 54.86 (1C, 11-C), 53.77 (1C, 10-C), 48.54 (1C, 7-C), 42.91 (1C, 3-C), 42.71 (1C, 4-C), 30.25 (3C, 12-C), 27.00 (1C, 5-C), 26.31 (1C, 6-C), 19.87 (1C, 8-C), 19.64 (1C, 9-C).

*1-((1S,4S)-3-((E)-ferrocenylmethylidene)-7,7-dimethyl-2-oxobicyclo[2.2.1]heptan-1-yl)-N-(tert-butyl)methanesulfonamide (**5**):*

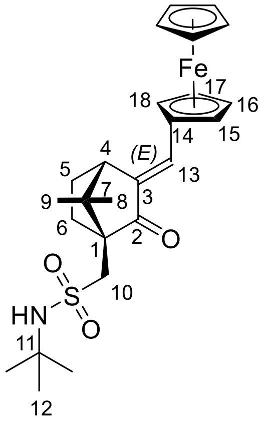

To a solution of 0.500 g (1.74 mmol) **3** and 0.372 g (1.74 mmol) ferrocenecarbaldehyde (**4**) in 25 ml dry toluene were added powdered KOH (0.195 g, 3.48) and a crystal of 18-crown-6. The mixture was refluxed for 3 h (TLC monitoring – DCM) and cooled. 30 ml Et_2O was added, washed with water and dried over anhydrous Na_2SO_4. The solvent was evaporated *in vacuo* and crude product was purified by column chromatography (80 g silica gel, mobile phase – a) DCM, b) DCM:MTBE = 10:1) to give 0.779 g (93%) pure **5** as red crystals. M.p. 133-134 °C. ^{1}H NMR (600.13 MHz, $CDCl_3$, 293 K): δ = 7.13 (s, 1H, 13-H), 5.65 (s, 1H, N<u>H</u>) 4.52 (m, 1H, 15-H), 4.51 (m, 1H, 18-H), 4.45 (m, 1H, 17-H), 4.43 (dt, 1H, 16-H, *J* = 2.5, 1.3 Hz), 4.15 (s, 5H, Cp), 3.58 (d, 1H, 10-H_a, *J* = 15.0 Hz), 3.06 (d, 1H, 10-H_b, *J* = 15.0 Hz), 2.93 (br d, 1H, 4-H, *J* = 4.0 Hz), 2.32 (m, 1H, 6-H_{exo}), 2.17 (tt, 1H, 5-H_{exo}, *J* = 11.4, 4.5 Hz), 2.03 (ddd, 1H, 6-H_{endo}, *J* = 14.1, 9.3, 4.8 Hz), 1.56 (m, 1H, 5-H_{endo}), 1.45 (s, 9H, 12-H), 1.05 (s, 3H, 9-H), 0.89 (s, 3H, 8-H). ^{13}C NMR (150.92 MHz, $CDCl_3$, 293 K): δ = 204.91 (1C, 2-C), 136.48 (1C, 3-C), 130.75 (1C, 13-C), 78.06 (1C, 14-C), 71.83 (1C, 15-C), 71.05* (1C, 16-C or 17-C), 71.03* (1C, 16-C or 17-C), 69.47 (5C, Cp), 68.91 (1C, 18-C), 58.68 (1C, 1-C), 54.89 (1C, 11-C), 54.12 (1C, 10-C), 48.92 (1C, 4-C), 48.28 (1C, 7-C), 30.30 (3C, 12-C), 27.81 (1C, 6-C), 25.62 (1C, 5-C), 20.74 (1C, 9-C), 18.89 (1C, 8-C). MS (CI) *m/z* (rel. int.): 484 (96, M+1), 483 (55, M), 428 (60), 411 (100), 347 (25). Anal. calcd. for $C_{25}H_{33}FeNO_3S$ (483.44): C, 62.11; H, 6.88; Fe, 11.55; N, 2.90; S, 6.63. Found: C, 62.19; H, 6.92; Fe, 11.50; N, 2.88; S, 6.67 %.

Synthesis of α-propargyl-ε-caprolactone

ε-Caprolactone (2.5 g, 21.9 mmol) was dissolved in anhydrous THF (10 mL) and the solution was added dropwise to a solution of lithium diisopropyl amide (12 mL, 24.1 mmol) in anhydrous THF (80 mL) pre-cooled at –78 °C under inert atmosphere. The reagents were stirred for 1 h at -78 °C before adding dropwise propargyl bromide (2.92 mL, 26.3 mmol) and hexamethylphosphoramide (5 mL). Next, the mixture was heated to -32 °C, stirred for additional 2 h and finally quenched with a saturated ammonium chloride solution. The liquids were evaporated; the resulting product was dissolved in diethyl ether and washed with saturated NaCl aqueous solution. The organic phase was dried over $MgSO_4$ and evaporated under reduced pressure. Silica gel column chromatography (heptane/EtOAc, 2/1) was used to purify the crude product. Finally, a colourless liquid monomer was obtained. Yield 70%.^{1}H-NMR ($CDCl_3$, 600 MHz): δ (ppm) = 4.11 (m, 2H, C*H*$_2$O), 2.56 (m, 1H, C(O)C*H*CH_2), 2.43 (m, 1H, C*H*$_2$-C≡C*H*), 2.30 (m,

1H, CH_2-C≡CH), 1.99 (t-d, 1H, C≡C*H*), 1.75-1.55 (m, 4H, CH_2CH_2CH_2O), 1.37 (m, 2H, C(O)CHCH_2).

Synthesis of copolymer

α,ω-Dihydroxy poly(α-propargyl-ε-caprolactone-co-ε-caprolactone)

ε-Caprolactone (2.71 g, 23.7 mmol, 90 eq.), α-propargyl-ε-caprolactone (0.4 g, 2.63 mmol, 10 eq.), butanediol (23.3 μL, 0.263 mmol, 1 eq.) and toluene (8 mL) were placed in a 50 mL two-neck round bottom flask under an inert atmosphere. The solution was degassed by argon flow for 20 min and $Sn(OTf)_2$ (55.0 mg, 0.132 mol, 0.5 eq.) was added, followed by further degassing for 20 min. The reaction mixture was stirred for 48 h at room temperature and then poured into cold methanol. The obtained polymer was filtered and dried in a vacuum oven. Yield 83%. ^{1}H-NMR(600 MHz, $CDCl_3$) δ (ppm)= 4.20-3.90 (m, 98H, -CH_2O-), 3.64 (t, 4H, -CH_2OH), 2.58-2.53 (m, 3H, C(O)C*H*CH_2), 2.48-2.45 (m, 3H, -C(O)CHCH_2), 2.42-2.38 (m, 3H, C(O)CHCH_2), 2.35-2.21 (m, 93H, -C(O)CH_2), 2.01 (s, 3H, -C≡C*H*), 1.80-1.50 (m, 196H, -CH_2-CH_2-CH_2-CH_2-O-), 1.49-1.30 (m, 98H, -CH_2-CH_2-CH_2-CH_2-O-), 1.27-1.17 (m, 4H, -CH_2CH_2-).

α,ω-Dihydroxy poly(α-cinnamyl-ε-caprolactone-co-ε-caprolactone)

Poly(α-propargyl-ε-caprolactone$_3$-*co*-ε-caprolactone$_{46}$) (1.0 g, M_n=5700 g.mol^{-1}, 0.175 mmol; 1 eq. of pendant functional groups), CuI (388.5 mg, 2.04 mmol, 4 eq.), cinnamyl azide (162.4 mg, 1.02 mmol, 2 eq.) were added into a two-neck round bottom flask under an inert atmosphere and degassed 3 times (vacuum-nitrogen cycles). After that, 5 mL of THF was added and the system was purged for 20 min with inert gas. Finally, degassed N,N-diisopropylethylamine (263.6 mg, 2.04 mmol, 4 eq.) was added and the reaction mixture was stirred for 24 h at 35°C. Then, 50 mL of THF was added and the reaction mixture was passed through a neutral aluminium oxide plug to remove the cupper salts. The mixture was then dissolved in a minimal amount of THF and precipitated into cold methanol. ^{1}H-NMR(600MHz, $CDCl_3$)) δ (ppm)= 7.4-7.15 (br m, 25H, Ar*H*), 4.2-3.9 (m, 98H, -CH_2O-), 3.66 (t, 4H, -CH_2OH), 2.4-2.18 (m, 98H, C(O)CH_2), 1.8-1.5 (m, 196H, -CH_2-CH_2-CH_2-CH_2-O-), 1.48-1.3 (m, 98H, -CH_2-CH_2-CH_2-CH_2-O-), 1.27-1.22 (m, 4H, -CH_2CH_2).

α,ω-Dibromo poly(α-cinnamyl-ε-caprolactone-co-ε-caprolactone)

α,ω-Dihydroxy poly(α-cinnamyl-ε-caprolactone$_3$-*co*-ε-caprolactone$_{46}$) (6 mol % cinnamyl units; 0.5 g, 0.088 mmol, 1 eq.) was dried under high vacuum for 3 h and dissolved in 8 mL of dry toluene in a 50 mL two-neck round bottom flask at room temperature under inert atmosphere. Triethylamine (35.6 mg, 0.352 mmol, 4 eq.) was added first, followed by dropwise addition of 2-bromoisobutiryl bromide (80.9 mg, 0.352 mmol, 4 eq.). The reaction mixture was stirred for 48 h at room temperature and the product was filtered by blue-stripe paper filter to remove HBr:Et_3N salt. Next, the solution was stirred on an activated carbon overnight and filtered by blue-stripe paper filter, concentrated on rotary evaporator, precipitated in 50 mL methanol, and finally dried thoroughly at 35 °C in the vacuum oven. ^{1}H-NMR (600MHz, $CDCl_3$): δ (ppm) = 7.45-7.15 (br m, 25H, Ar*H*), 4.17 (t, 4H, -CH_2O-), 4.15-3.73 (m, 98H, -CH_2O-), 2.4-2.18 (m, 98H, C(O)CH_2), 1.92 ppm (s, 12H, -C-CH_3), 1.75-1.5 (m, 196H, -CH_2-CH_2-CH_2-CH_2-O-), 1.45-1.3 (m, 98H, -CH_2-CH_2-CH_2-CH_2-O-), 1.27-1.22 (m, 4H, -CH_2CH_2-).

Poly(α-cinnamyl-ε-caprolactone-co-ε-caprolactone) macroreagent comprising two azide end-groups

α,ω-Dibromo poly(*α*-cinnamyl-ε-caprolactone$_3$-*co*-ε-caprolactone$_{46}$) (6 mol% cinnamyl units; 0.3 g, 0.053 mmol, 1 eq.) was dried for 3 h under high vacuum and dissolved in 3 mL of anhydrous DMF in a 50 mL two-neck round bottom flask at room temperature under inert atmosphere. NaN_3 (71.5 mg, 1.1 mmol, 20 eq.) was added, temperature was increased, and the reaction mixture was stirred at 50 °C for 48 h. The solution was concentrated under reduced pressure on a rotary evaporator, diluted with 40 mL of CH_2Cl_2, washed with 50 mL of water and 2x50 mL portions of brine. Next, the collected aqueous phase was extracted with 40 mL of CH_2Cl_2; the obtained organic solution was dried with $MgSO_4$ and then CH_2Cl_2 was evaporated under vacuum. The product was re-dissolved in 3 mL of CH_2Cl_2 and precipitated into 30 mL of cold methanol. The copolymer was recovered by filtration and dried in vacuum oven. ^{1}H-NMR(600MHz, $CDCl_3$): δ (ppm)= 7.45-7.2 (br m, 25H, Ar*H*), 4.17 (t, 4H, -CH_2O-), 4.15-3.85 (m, 98H, -CH_2O-), 2.10-2.40 (m, 98H, C(O)CH_2), 1.8-1.5 (m, 196H, -CH_2-CH$_2$-CH_2-CH$_2$-O-), 1.46 (s, 12H, -C-CH_3), 1.42-1.35 (m, 98H, -CH$_2$-CH_2-CH$_2$-CH$_2$-O-), 1.35-1.3 (m, 4H, -CH_2CH_2-).

Mono-alkyn functional poly(ethylene oxide) macroreagent

Polyethylene glycol monomethyl ether (1.25 g, 0.25 mmol, 1 eq.) was dried by azeotropic distillation with toluene and then dissolved in dry dichloromethane (20 mL) under inert atmosphere. Pentynoic acid (49.1 mg, 0.5 mmol, 2 eq.), EDC (95.9 mg, 0.5 mmol, 2 eq.) and DMAP (30.5 mg, 0.25 mmol, 1 eq.) were subsequently added to the solution at room temperature. The reaction mixture was stirred for 24 h, diluted with 60 mL of CH_2Cl_2 and extracted with 2x75 mL of distilled water. The organic phase was dried over $MgSO_4$, filtered and finally CH_2Cl_2 was evaporated. The product was re-dissolved in 10 mL of CH_2Cl_2 and precipitated in 100 mL of chilled diethyl ether. Yield 90%.^{1}H-NMR ($CDCl_3$, δ ppm): 4.27 (t, 2H -CH_2OC(O)- group), 3.80–3.50 (m, 450H, -OCH_2CH_2O-), 3.38 (s, 3H,CH_3O-PEO), 2.57–2.52 (m,2H, -CH_2-CH$_2$–C≡CH group), 2.52–2.47 (m, 2H, -CH$_2$-CH_2–C≡CH group), 1.99 (t, 1H, –C≡C*H* group).

Poly(ethylene oxide)-b-poly(α-cinnamyl-ε-caprolactone-co-ε-caprolactone)-b-poly(ethylene oxide) copolymer

Azide-bifunctional poly(*α*-cinnamyl-ε-caprolactone$_3$-*co*-ε-caprolactone$_{46}$) (6 mol% of cinnamyl units; 0.250 g, 0.0439 mmol, 1 eq.), mono-alkyne functional PEO$_{113}$ (0.878 g, 0.1756, 4 eq.) and CuBr (125.9 mg, 0.878 mmol, 20 eq.) were added into 50 mL two-neck round bottom flask under an inert atmosphere followed by three freeze-pump-thaws degassing cycles. Then, anhydrous DMF (6 mL) was added via a syringe and the solution was purged with argon under stirring for 20 min. PMDETA (182.7 mg, 1.054 mmol, 24 eq.) was added via a syringe and the solution was purged additionally for 20 min with argon. The reaction mixture was stirred for 24 h at 35 °C. Next, 60 mL of dioxane were added and the solution was passed through silica gel column for removing cupper salts. The filtered product was dissolved in 50 mL of acetone and 100 mL of water were added dropwise. The organic solvent was evaporated on a rotary evaporator and the remaining in the aqueous phase copolymers was purified by ultrafiltration (membrane MW cut off 10 000 g.mol^{-1}) and dialysis against acetone/water

(1/1 v./v.) mixture (membrane MW cut off 6 000 g.mol^{-1}). Finally, the product was collected by freeze-drying. ^{1}H-NMR (600MHz, $CDCl_3$): δ (ppm)= 7.68 (s, 2H, triazole ring), 7.4-7.2 (br m, 25H, Ar*H*), 4.21 (t, 4H, -CH_2O-), 4.12 (t, 4H, -CH_2O-), 4.1-4.0 (m, 98H, -CH_2O-), 3.75-3.5 (m, 900H, CH_2-CH_2-O-), 3.36 (s, 6H, CH_3-O-), 3.12 (t, 4H, -CH_2-), 2.82 (t, 4H, -CH_2-), 2.35-2.25 (m, 98H, C(O)CH_2), 1.92 (s, 12H, -C-CH_3), 1.7-1.5 (m, 196H, -CH_2-CH_2-CH_2-CH_2-O-), 1.45-1.3 (m, 98H, -CH_2-CH_2-CH_2-CH_2-O-), 1.3-1.25 (m, 4H, -CH_2CH_2-).

TABLES

Table S1. Release kinetic models for DK-164-NP

Formulation	Zero order	First order	Higuchi model	Korsmeyer-Peppas
	R^2	R^2	R^2	R^2
DK-164-NP	0.7383	0.7675	0.976	**0.996**

FUGURES

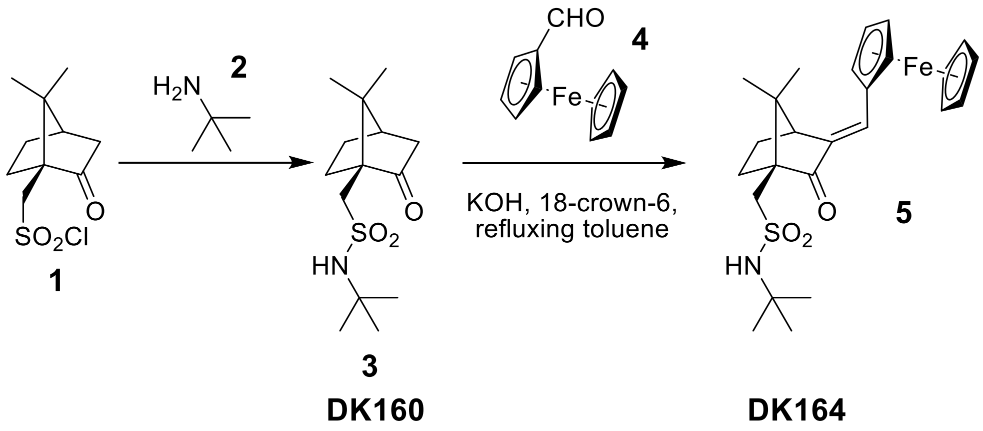

Figure S1. Schematic representation of the synthesis of DK164.

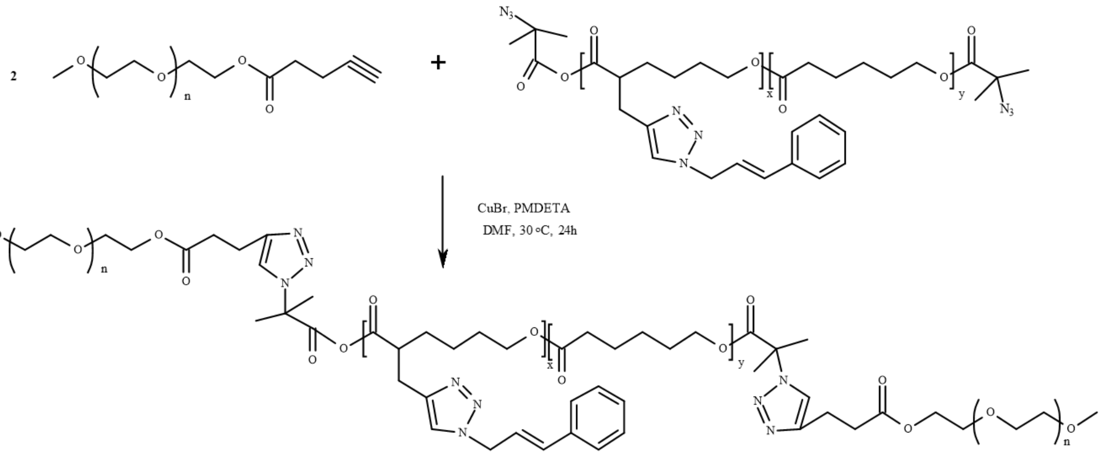

Figure S2. Schematic representation of the synthesis of PEO$_{113}$-*b*-P(CyCL$_3$-*co*-CL$_{46}$)-*b*-PEO$_{113}$.

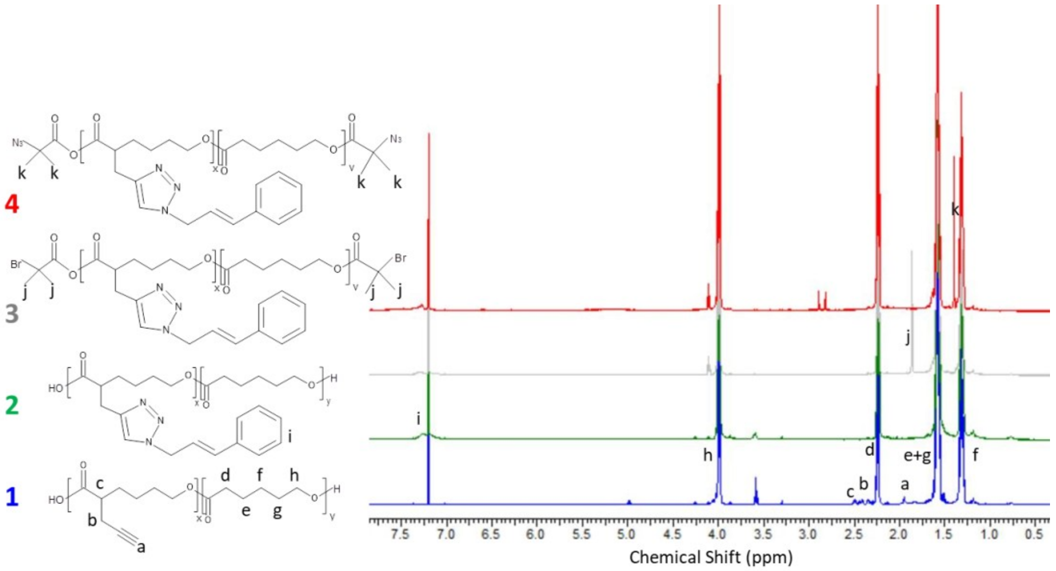

Figure S3. ^{1}H-NMR spectra of: (1) α,ω-dihydroxy poly(α-propargyl-ε-CL$_3$-*co*-ε-CL$_{46}$); (2) α,ω-dihydroxy poly(α-cinnamyl-ε-CL$_3$-*co*-ε-CL$_{46}$); (3) α,ω-dibromo poly(α-cinnamyl-ε-CL$_3$-*co*-ε-CL$_{46}$); and (4) α,ω-azide terminated poly(α-cinnamyl-ε-CL$_3$-*co*-ε-CL$_{46}$) in CDCl$_3$.

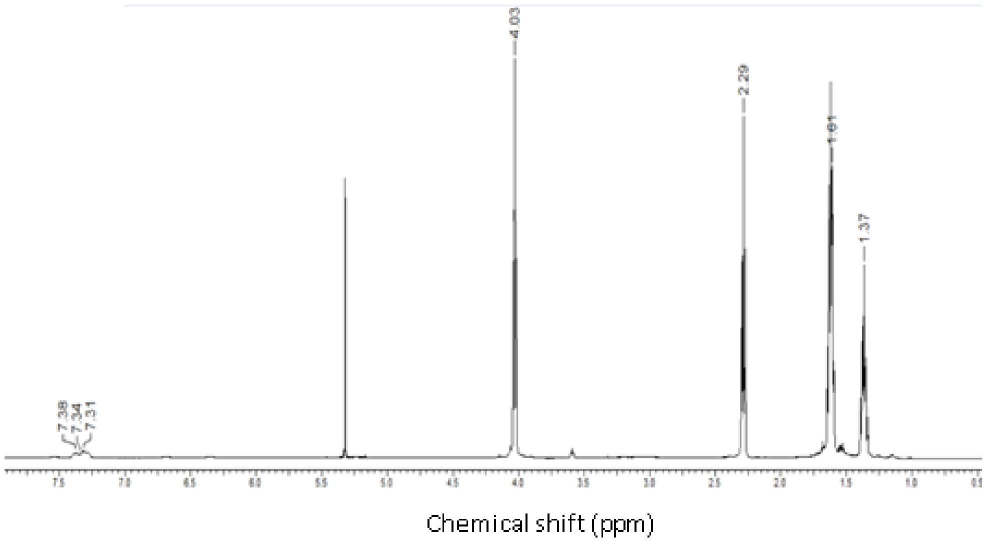

Figure S4. ^{1}H-NMR spectrum of PEO_{113}-*b*-P($CyCL_3$-*co*-CL_{46})-*b*-PEO_{113} in $CDCl_3$.

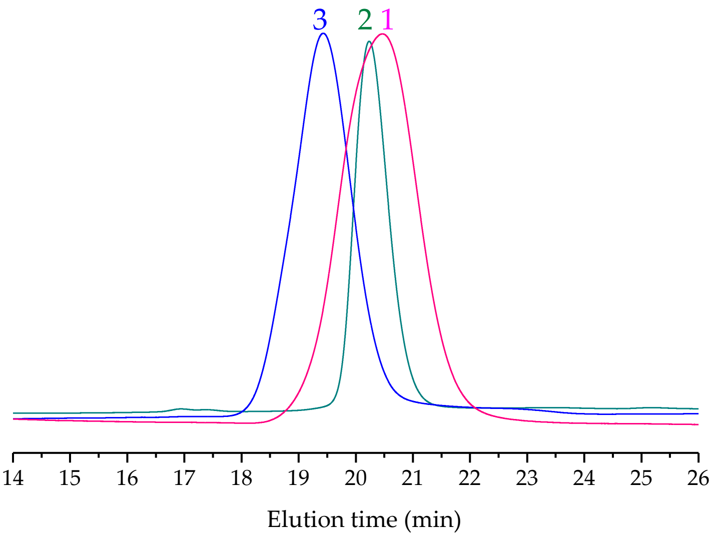

Figure S5. GPC chromatograms of: (1) N_3-P($CyCL_3$-*co*-ε-CL_{46})-N_3, (2) PEO_{113}-C≡CH macroreagents, and (3) PEO_{113}-b-P($CyCL_3$-co-CL_{46})-b-PEO_{113} triblock copolymer.

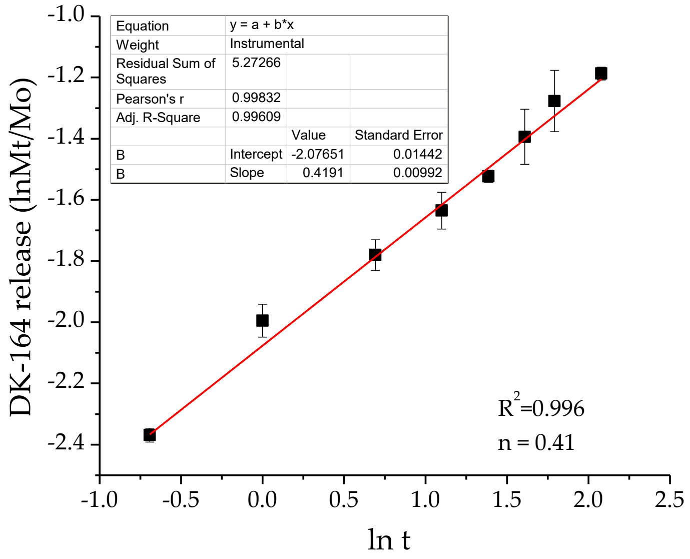

Figure S6. Plot of the released amount of DK-164 versus ln t (Korsmeyer-Peppas model).

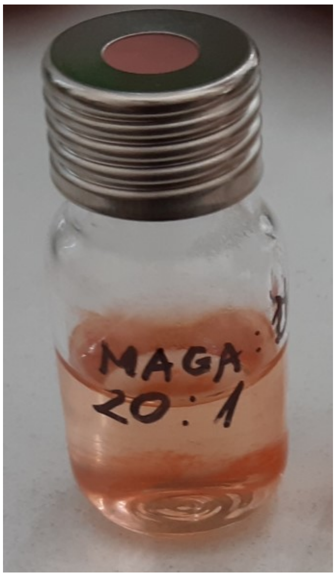

Figure S7. Digital picture of water-based sample containing DK164 and PEO_{113}-*b*-PCL_{35}-*b*-PEO_{113}, taken 24 h after loading the drug into the micelles.

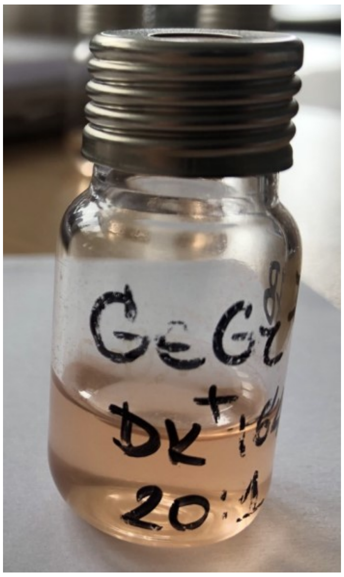

Figure S8. Digital picture of aqueous colloid of DK164 and PEO_{113}-b-P($CyCL_{3}$-co-CL_{46})-b-PEO_{113}, taken 24 h after loading the drug into the micelles.

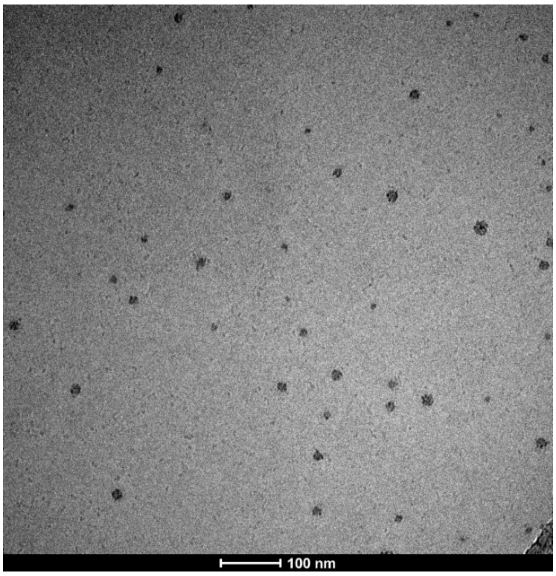

Figure S9. Cryo-TEM micrograph of PEO_{113}-b-P($CyCL_{3}$-co-CL_{46})-b-PEO_{113} micelles in water.
